# Impact of storage conditions on the quality of nucleic acids in paraffin embedded tissues

**DOI:** 10.1371/journal.pone.0203608

**Published:** 2018-09-07

**Authors:** Daniel Groelz, Christian Viertler, Daniela Pabst, Nadine Dettmann, Kurt Zatloukal

**Affiliations:** 1 QIAGEN GmbH, Research and Development, Hilden, Germany; 2 Institute of Pathology, Medical University of Graz, Graz, Austria; Centro Nacional de Investigaciones Oncologicas, SPAIN

## Abstract

RNA and DNA analyses from paraffin-embedded tissues (PET) are an important diagnostic tool for characterization of a disease, exploring biomarkers and treatment options. Since nucleic acids from formalin-fixed and paraffin-embedded (FFPE) tissue are of limited use for molecular analyses due to chemical modifications of biomolecules alternate, formalin-free fixation reagents such as the PAXgene Tissue system are of evolving interest. Furthermore, biomedical research and biomarker development critically relies on using long-term stored PET from medical archives or biobanks to correlate molecular features with long-term disease outcomes. We therefore performed a comparative study to evaluate the effect of long term storage of FFPE and PAXgene Tissue-fixed and paraffin-embedded (PFPE) tissue at different temperatures on nucleic acid stability and usability in PCR. Matched FFPE and PFPE human tissues from routine clinical setting or rat tissues from a highly controlled animal model were stored at room temperature and 4°C, as well as in case of animal tissues frozen at -20°C and -80°C. RNA and DNA were extracted in intervals for up to nine years, and examined for integrity, and usability in quantitative RT-PCR (RT-qPCR) or PCR (qPCR) assays. PET storage at room temperature led to a degradation of nucleic acids which was slowed down by storage at 4°C and prevented by storage at -20°C or -80°C. Degradation was associated with an amplicon length depending decrease of RT-qPCR and qPCR efficiency. Storage at 4°C improved amplifiability in RT-qPCR and qPCR profoundly. Chemically unmodified nucleic acids from PFPE tissue performed superior compared to FFPE tissue, regardless of storage time and temperature in both human and rat tissues. In conclusion molecular analyses from PET can be greatly improved by using a non-crosslinking fixative and storage at lower temperatures such as 4°C, which should be considered in prospective clinical studies.

## Introduction

Paraffin embedded tissue (PET) blocks are generated in huge quantities in the context of histopathological diagnostics and stored in large medical archives or biobanks. These archives constitute a very valuable resource for retrospective studies in basic biomedical research as well as for development of new diagnostics or therapies. Archiving is done mainly at room temperature and it is well established that FFPE tissues stored for decades can still be used for histological examination. In addition, despite increasing levels of degradation, nucleic acids and proteins can be extracted from archived FFPE tissues and are open to some extent for molecular analyzes [1–3]. However, there is lack of systematic evaluation of the effect of long-term storage conditions on quality and integrity of biomolecules in FFPE tissues. Moreover, such studies do not exist at all for PET tissues which were fixed with non-formaldehyde-based fixatives.

Fixation of tissues with neutral buffered formalin (NBF), a 4% aqueous formaldehyde solution, has become the standard method for preservation of tissue since introduction of formaldehyde into pathology work in the late 19^th^ century [4]. Beside excellent preservation of tissue morphology, contrariwise formaldehyde leads to crosslinking and chemical modification of nucleic acids and proteins. In a complex reaction cascade which is still not fully understood, addition of methylol groups and formation of methylene bridges eventually leads to crosslinks between proteins, and proteins and nucleic acids [5]. These chemical modifications of biomolecules can lead to sequence artefacts in DNA [6], as well as reduced efficiency of cDNA synthesis from RNA which can lead to gene to gene variations in RT-qPCR and microarray based gene expression analysis [7]. In addition, formalin was recently reclassified in the EU (category 2/3 to category 1B/2, regulation 605/2014) because of its potential carcinogenicity and has now to be marked as carcinogenic and mutagenic. Based on this reclassification the use of formaldehyde becomes increasingly restricted.

In recent years a couple of formalin free, less toxic, “molecular friendly” tissue preservatives were suggested in order to improve the quality of nucleic acids from PETs [8–12]. However for different reasons none of these has yet gained broader acceptance. With upcoming personalized medicine applications getting entry into clinical routine and pathology diagnostics, i.e. identification of driver mutations in combination with targeted therapy, there is a renewed interest in less toxic tissue fixation methods which do not chemically modify biomolecules.

PAXgene Tissue, a non-crosslinking, alcohol-based tissue fixation and stabilization system was developed and validated within the EU financed framework programe 7 (FP7) project SPIDIA [13]. It was tested for its characteristics to preserve tissue morphology equally to NBF [14] but to outperform crosslinking fixatives with regard to preservation of nucleic acids [13–15], proteins [16] and phosphoproteins [17]. Evaluation of blinded, randomized, international ring trials with PFPE and matched FFPE cancer specimen came to the conclusion that the quality of histo- and cytomorphological features of PFPE specimen allows use of PAXgene Tissue for routine morphological diagnosis of colon [18] and breast cancer tissue (Viertler et al., in preparation). Further studies conducted outside SPIDIA confirmed compatibility of PAXgene Tissue treated tissues with commonly used methods such as immunohistochemistry (IHC) [19–21] and fluorescence *in situ* hybridization (FISH) [22,23] as well as with molecular biology applications including RT-qPCR [20,21,24–26], NGS technologies and genome-wide DNA methylation analysis [27–29] and metabolomic and proteomic analysis [30].

The aim of this long-term study was to systematically evaluate the effect of storage time and temperature on RNA and DNA quality in PET tissues fixed either with NBF or PAXgene Tissue. For this purpose human tissues from a routine clinical setting as well as tissues from an animal model processed under highly standardized preanalytical conditions were collected during the EU funded SPIDIA project. Human tissues were stored at room temperature and at 4°C for up to seven years. Animal tissues were stored at 22°C, 4°C, -20°C and -80°C for up to nine years. RNA and DNA were extracted from PET tissues stored under the different conditions, examined for integrity in microcapillary electrophoresis, and amplified in RT-qPCR or qPCR assays.

## Materials and methods

### Human tissue collection, preservation, processing and storage

In total eight different cases of human malignant (liver, soft tissue) and non-malignant tissues (stomach, duodenum, liver) were collected and divided into equal aliquots. These aliguots were either snap-frozen in methyl butane cooled by liquid nitrogen (Cryo), NBF (for 3 hours, 24 hours, and up to 120 hours), or fixed in PAXgene Tissue Fix (for 3 hours, 24 hours, and up to 120 hours) and then transferred into PAXgene Tissue Stabilizer (for 24 hours and up to 120 hours) at room temperature (PreAnalytiX GmbH, Hombrechtikon, Switzerland) to simulate variability of fixation, transport or storage times in clinical routine workflows and to generate matched NBF-fixed, PAXgene Tissue-fixed and Cryo tissues per case. After stepwise dehydration in 70%, 80%, 90%, and 99% ethanol followed by isopropanol and xylene, the PAXgene Tissue-fixed and NBF-fixed tissues were embedded in low-melting paraffin as described elsewhere [13]. Corresponding PFPE and FFPE blocks were stored in parallel in the dark at 4°C and at room temperature. All tissues were obtained with written informed patient consent and the study was approved by the Ethics Committee of the Medical University of Graz, Austria (reference number 20–066).

### Rat tissue collection, preservation, processing and storage

Rats (*rattus norvegicus*) were raised to a weight of approximately 500 g before being sacrificed. Organs (liver, kidney, spleen, intestine, lung) were removed within 5 min of sacrifice. To generate matched tissues, adjacent, equally sized tissues no larger than 15 × 15 × 4 mm were grossed from each organ and directly fixed in NBF (for 24 hours) or fixed in PAXgene Tissue Fix (for 2–4 hours) and then transferred into PAXgene Tissue Stabilizer (for up to 72 hours) at room temperature to simulate transport or storage times in clinical settings. For cryo-preservation, tissues were snap-frozen in liquid nitrogen and stored at -80°C. After stepwise dehydration in ethanol, followed by isopropanol and xylene, the PAXgene Tissue-fixed and NBF-fixed tissues were embedded in low-melting paraffin as described elsewhere [15]. PFPE and FFPE blocks were stored in parallel in the dark at 22°C, 4°C, -20°C and -80°C.

Rats were maintained and sacrificed by CO2 asphyxiation at ZETT (Zentrale Einrichtung für Tierforschung und wissenschaftliche Tierschutzaufgaben, Düsseldorf, Germany), the central institution of the Heinrich Heine university of Düsseldorf, licensed to raise and sacrifice animals. Animal care was done by trained veterinary clinicians from ZETT only and in accordance with the German protection of animals act and EU guideline 2010/63. None of the authors were involved in handling and sacrificing the animals.

### RNA extraction and integrity

RNA from human tissues was extracted from 5 μm sections (10–20 sections per tissue) using *RNeasy FFPE Kit* (QIAGEN GmbH, Hilden, Germany) for FFPE, the *PAXgene Tissue RNA Kit* (PreAnalytiX) for PFPE, or the Invitrogen TRIzol procedure (Thermo Fisher Scientific, Wilmington, DE) for 20–70 mg of snap-frozen tissues according to the manufacturer’s instructions.

RNA from rat FFPE and PFPE tissue blocks was extracted from 10 μm sections (3–5 sections per tissue) with *RNeasy FFPE and PAXgene Tissue RNA Kits* respectively, and with the *RNeasy Mini Kit* (QIAGEN) from 10 mg of snap-frozen reference tissue. Before selecting sections for RNA extraction, at least 3 sections (10 μm) were cut from the PET block and discarded.

For human and rat tissues, RNA concentration in the eluates was determined using a NanoDrop ND-1000 spectrophotometer (Thermo Fisher Scientific, Wilmington, DE), and electropherograms were obtained using an Agilent 2100 Bioanalyzer platform with an Agilent RNA 6000 Nano Kit (Agilent Technologies, Santa Clara, CA). Agilent 2100 Expert software version B.02.03.SI307 was used to calculate the RNA integrity number.

### Quantitative RT-PCR (RT-qPCR)

Human RNA was analyzed by reverse transcription of 1 μg RNA in a total reaction volume of 25μl into cDNA using High-Capacity cDNA Reverse Transcription Kit (ThermoFisher Scientific, Germany) according to the manufacturer’s instructions. Four microliters of 1:32 cDNA dilutions served as template for PCR using Power SYBR Green PCR Master Mix (Thermo Fisher Scientific, Wilmington, DE). Fragments of human *GAPDH* with amplicon lengths of 71, 153, 200, 277 and 323 base pair (bp) were amplified as published previously [13]. All samples and controls were analyzed in triplicates for 45 cycles of PCR using a MicroAmp fast 96-well reaction plate format on an ABI 7900 Real-Time PCR System (Thermo Fisher Scientific, Wilmington, DE). Results were analyzed using ABI SDS software version 2.3. Data points containing nonspecific products, as identified by melting curve analysis, were excluded (https://dx.doi.org/10.17504/protocols.io.qrgdv3w).

Rat tissue RNA eluates were analyzed with specific rat *ACTB* gene RT-qPCR assays. Performance in dependence of amplicon lengths was investigated with custom-designed rat *ACTB* gene primer sets using 10 ng RNA in one-step RT-PCR assays on a Rotor-Gene Q using QuantiTect® SYBR Green RT-PCR Kits (QIAGEN) according to manufacturer´s instructions to amplify 109 to 438 bp fragments. All samples and controls were analyzed in triplicates for 40 cycles of PCR using a 72 well format on a Rotor-Gene Q PCR-Cycler (QIAGEN). Results were analyzed using Rotor Gene Q Series Software 2.3.1. Data points containing nonspecific products, as identified by melting curve analysis, were excluded (https://dx.doi.org/10.17504/protocols.io.qrfdv3n).

Statistical analyses were carried out using GraphPad Prism 7 software (version 7.03), one-way ANOVA multiple comparison (nonparametric) to determine after which period of time performance differences between PET blocks stored at 4°C or room temperature becomes statistically relevant.

### DNA extraction and integrity

Genomic DNA from rat FFPE and PFPE tissue blocks was extracted from 3–5x 10 μm sections using the *PAXgene Tissue DNA Kit* (PreAnalytiX) for PFPE, the *QIAamp DNA FFPE Tissue Kit* (QIAGEN) for FFPE or the *DNeasy Tissue Kit* (QIAGEN) for 10 mg of cryo-preserved tissue according to the manufacturer instructions. DNA concentration in the eluates was measured with a NanoDrop^TM^ 2000 spectrophotometer (ThermoFisher) and confirmed on a Qubit® 2.0 Fluorometer with Qubit® dsDNA Assay (ThermoFisher). Integrity was assessed on Agilent 4200 TapeStation system with genomic DNA Analysis ScreenTape assay (Agilent Technologies).

### Quantitative PCR (qPCR)

For assessment of amplification performance 10ng of genomic DNA from PFPE and FFPE tissues were amplified with custom-designed rat *ACTB* gene primer sets (metabion GmbH) and QuantiTect® SYBR Green PCR Kits (QIAGEN) according to manufacturer´s instructions to amplify 271 to 747 bp fragments. All samples and controls were analyzed in triplicates for 40 cycles of PCR using a 72 well format on a Rotor-Gene Q PCR-Cycler (QIAGEN). Results were analyzed using Rotor Gene Q Series Software 2.3.1. The following primers were used to amplify amplicons with 271, 523, 650 and 747 bp lengths: 271 bp Rn_ACTB DNA-for1 5´-CTTGTGGCTTTAGGAGCTTGAC-3´ and Rn_ACTB DNA-rev1 5´- ACGCTCGGTCAGGATCTTCATG-3´; 523 bp Rn_ACTB DNA-for2 5´-TCGATCGCCTTTCTGACTAGG-3´ and Rn_ACTB DNA-rev1; 650 bp Rn_ACTB DNA-for2 and Rn_ACTB DNA-rev2 5´-TCTTCTCCAGGGAGGAAGAGGATG-3´; 747 bp Rn_ACTB DNA-for3 5´-CTTCTGCCATTCTCCCATAGG and Rn_ACTB DNA-rev2. Data points containing nonspecific products, as identified by melting curve analysis, were excluded (https://dx.doi.org/10.17504/protocols.io.qnidvce).

## Results

### RNA integrity in PET blocks as a function of storage temperature and time

**Fig 1:**
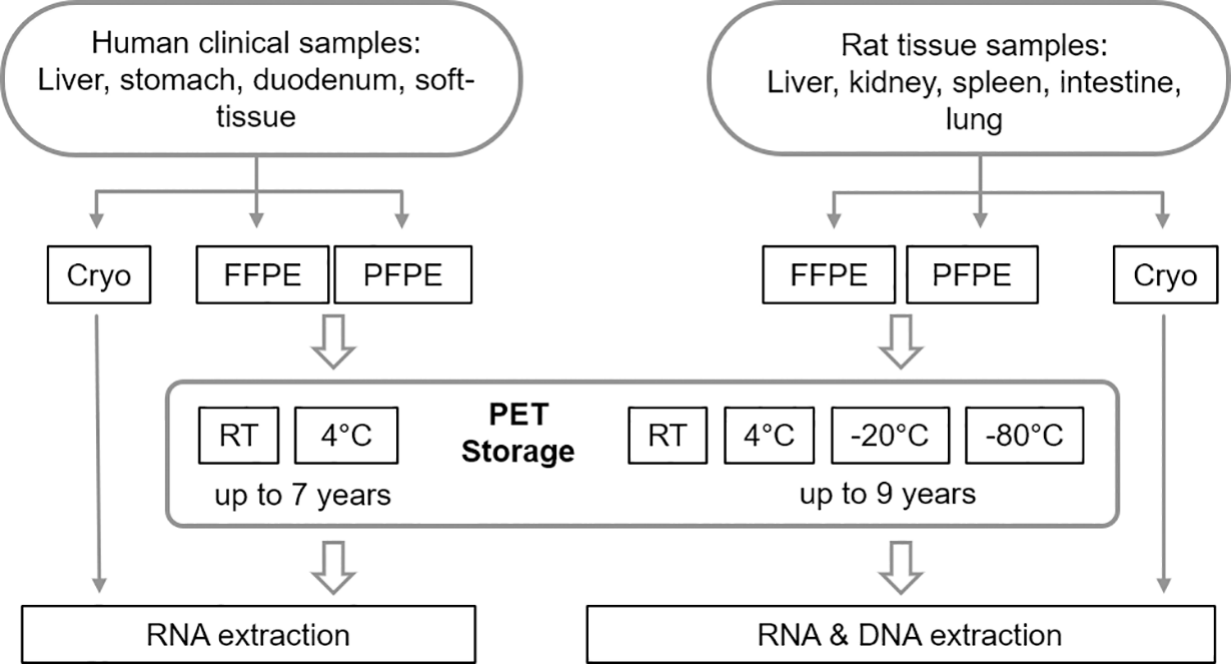
Schematic overview of the study setup. Human clinical or rat tissues were freshly collected, aliquoted and either directly snap-frozen (Cryo), or fixed in 4% neutral buffered formaldehyde (NBF) or in PAXgene Tissue Fix and Stabilizer. Paraffin embedded tissue blocks (PET) were stored under controlled temperatures for up to 7 years in case of human and up to 9 years in case of rat tissues until RNA and DNA extraction.

Corresponding aliquots of different malignant and non-malignant clinical human tissues (liver, gastrointestinal, soft tissue; S1 Table) were fixed in NBF or with the PAXgene Tissue system, embedded in paraffin and stored at room temperature or refrigerated at 4°C. At defined time points RNA was isolated from sections of FFPE and PFPE tissue and examined for integrity and usability in RT-qPCR assays. In order to exclude the influence of clinical workflow variances, such as warm and cold ischemia, the same approach was repeated with an animal rat model. Rat tissues (liver, kidney, spleen, intestine, lung) were collected, processed and stored under highly controlled preanalytical conditions. RNA and DNA was extracted, analyzed for yield and integrity, and used in RT-qPCR and qPCR assays, respectively (Fig 1).

**Fig 2:**
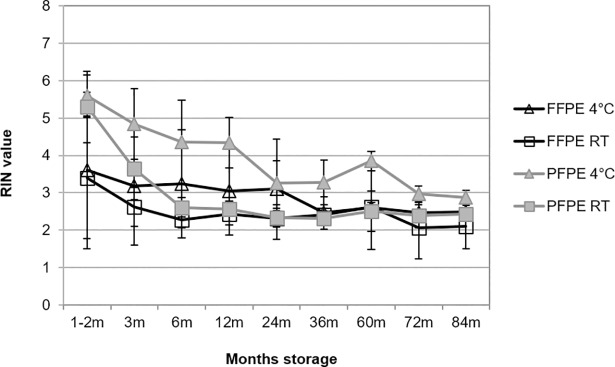
RNA integrity from human PET blocks decreases depending on storage temperature. Analysis of RNA integrity number (RIN) was performed from human PFPE and FFPE tissues using an Agilent Bioanalyzer. PET blocks of different tissue types were stored at room temperature (RT) and 4°C prior to RNA extraction. RNA was extracted from 8 different cases including four different tissue types (soft tissue, stomach, duodenum, liver) at several time points for up to 84 months (m) of storage (S1 Table). For each time point, fixation method and storage condition the mean RIN value with standard deviation was calculated (total RIN values n = 411).

**Fig 3:**
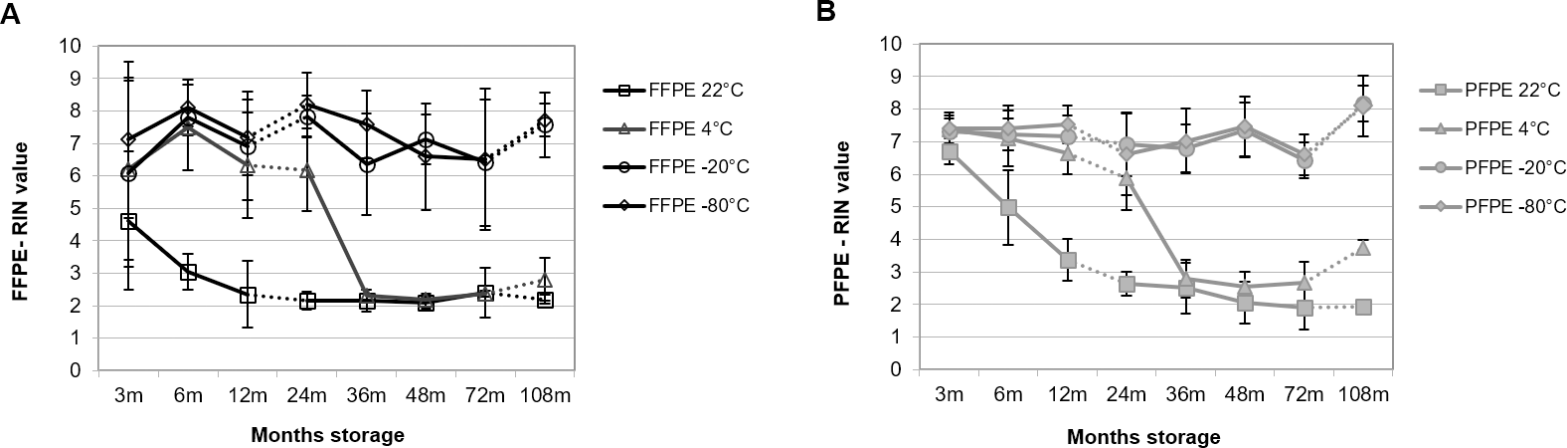
RNA integrity from rat PET blocks decrease depending on storage temperature. For each of five different tissue types (liver, kidney, spleen, lung and intestine) and four different temperatures (22°C, 4°C, -20°C, and -80°C) 3 blocks of FFPE and PFPE rat tissue were stored. RNA was extracted from block 1 after 3, 6 and 12 months (m), from block 2 after 24, 36, 48 and 72 m and from block 3 after 108 m of storage. Mean RNA integrity number (RIN) values with standard deviation from FFPE **(A)** and PFPE tissue **(B)** are shown for triplicate extractions from five different tissue types for each fixation method, storage time point and temperature (total RIN values n = 942). Dotted line indicates block change.

In total 411 human and 942 rat RNA eluates from PET blocks were analyzed. Thereby a correlation was found between RNA integrity and PET storage temperature for both, human tissues collected during routine clinical workflows (Fig 2) and rat tissue collected and processed under most stringently standardized preanalytical conditions (Fig 3). RNA extraction from human specimen started after 1 to 2 months of storage and RIN values varied between 2–6, depending on fixation type and case as indicated by the standard deviations (SD). For different fixation times in human samples no impact on RIN values were observed. Initially the average RIN value was 3.6 and 3.4 for FFPE and 5.6 and 5.3 for PFPE, at 4°C storage and room temperature, respectively. When stored at room temperature RNA integrity markedly declined already within the first 6 months and finally reached a bottom line for both FFPE and PFPE tissue with a RIN value of about 2–3. Degradation kinetics was slowed down but not prevented by storage at 4°C (Fig 2).

In case of RNA isolated from the animal model RIN values from PET were consistently higher compared to human PET (Fig 3). Variation across different tissues was higher for FFPE (Fig 3A) as compared to PFPE tissues (Fig 3B), indicated by larger SD. Storage of PET at room temperature (22°C) led to degradation of RNA in both PFPE and FFPE tissues. Similar to human tissues this degradation started directly after storage and RIN values declined to a bottom line of about 2–3 after 24 months. Also in rat PETs RNA degradation was slowed down when blocks were stored at 4°C, resulting in RIN 2–3 after 36 months. However, a difference of 0.5–2 RIN values still remained between tissues stored at 4°C versus 22°C, even after nine years of storage. In contrast freezing PET blocks at -20°C or -80°C prevented RNA from degradation in both rat FFPE and PFPE tissues.

### Improved RT-qPCR-performance of RNA from PET blocks stored at lower temperatures

**Fig 4:**
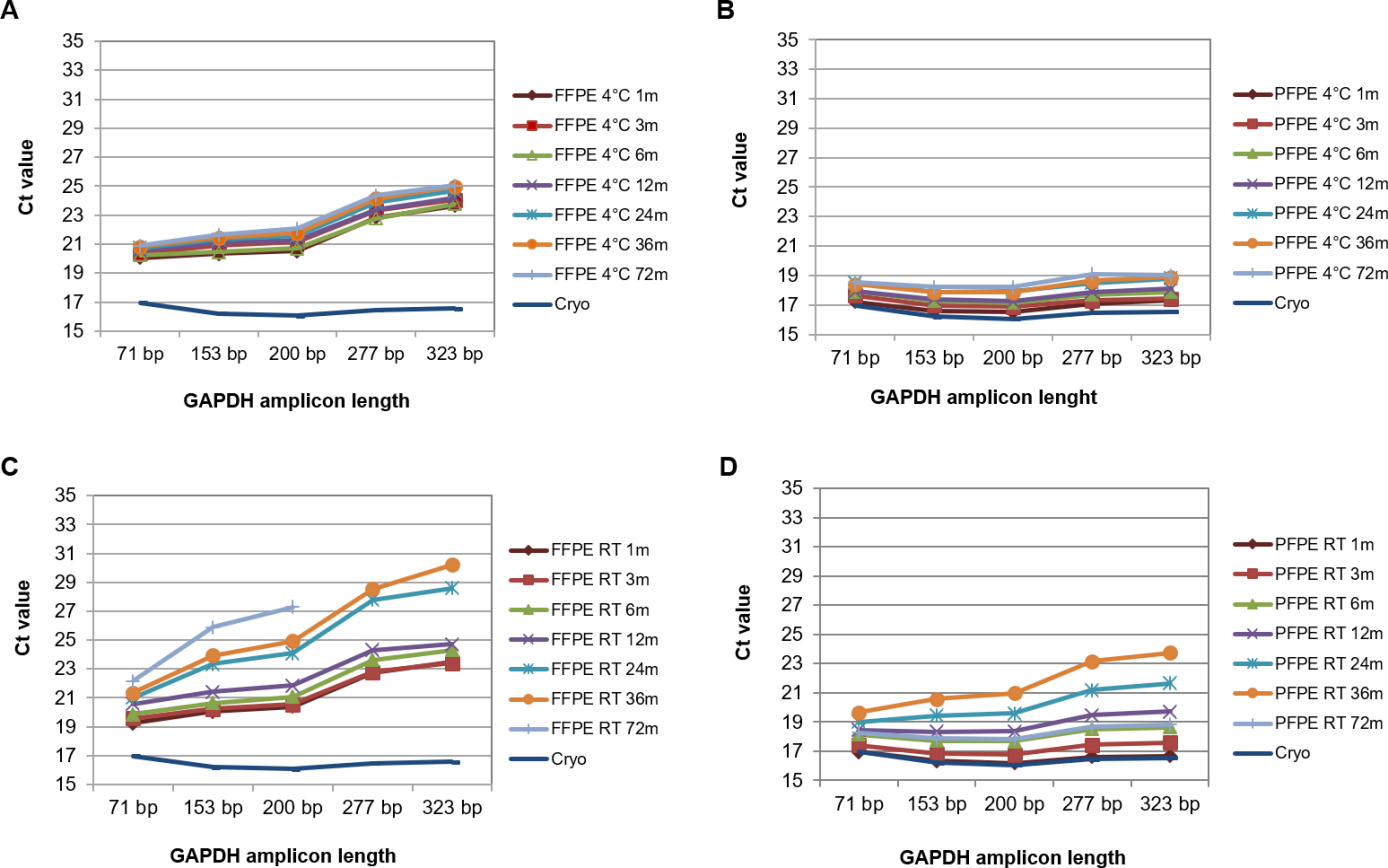
RT-qPCR-performance is independent from storage time in PET blocks stored at 4°C. Human FFPE (A and C) and PFPE (B and D) tissue (case #1, soft tissue) were stored at 4°C (A and B) or at room temperature (C and D) for up to 6 years prior to RNA extraction. Amplification of human GAPDH gene fragments ranging from 71 to 323 base pairs (bp) was performed in SYBR-Green RT-qPCR assays. Missing data points represent no specific amplification possible.

In order to test whether decreasing RNA integrity during storage affected performance in downstream applications, RT-qPCR was performed with sets of SYBR-Green assays amplifying human *GAPDH* or rat *ACTB* fragments with different amplicon lengths. In order to avoid alterations due to different fixation times in human samples, only samples fixed for 24 hours (except for 3 hours fixation in case 6) were analyzed. In Fig 4 RT-qPCR results are shown for a soft tissue sarcoma case (case #1, S1 Table) including RNA from matched snap-frozen material as reference. Five different fragments (from 71 bp to 323 bp) of the human *GAPDH* gene were amplified. In case of FFPE tissues increasing amplicon-length resulted in ascending cycle threshold (Ct) values, differing from the cryo-reference by at least 2 and up to 9 Ct for tissues stored at 4°C and up to 14 Ct values for tissues stored at room temperature (Fig 4A and Fig 4C). Assuming optimal PCR efficiency, a decrease of Ct values by one is equivalent with doubling the amount of target gene fragments, which are accessible by PCR. Therefore an increase of 2, 9 and 14 corresponded to a reduction to 25%, 0.195%, and 0.006% respectively (S2 Table shows correlation between increase of Ct value and decrease in number of target, amplifiable gene fragments) [31]. Despite the observed decreasing RNA integrity, storage time at 4°C had only minor effect on Ct values. The differences of Ct values between FFPE tissues stored for one month compared to FFPE tissues stored for 72 months was consistently low, with about 1–2 Ct (Fig 4A) for all assays. In contrast storage time of FFPE tissues stored at room temperature had a much larger effect on Ct values; Ct values increased here by 3 to 8 depending on storage time and amplicon lengths (Fig 4C).

RT-qPCR values generated with RNA from PFPE tissues were 2–7 Ct lower compared to corresponding FFPE tissues (Fig 4B and Fig 4D). However, similar to FFPE tissues, storage time had a negative effect on amplifiability when PFPE tissues were stored at room temperature, while storage at 4°C minimized this trend. At 4°C Ct values between PFPE tissues stored for 1 month and 72 months differed by only about 2 (Fig 4B). This difference increased to 3 Ct for the smallest (71 bp) and up to 7 Ct for the largest (323 bp) fragment in case of room temperature storage (Fig 4D).

**Fig 5:**
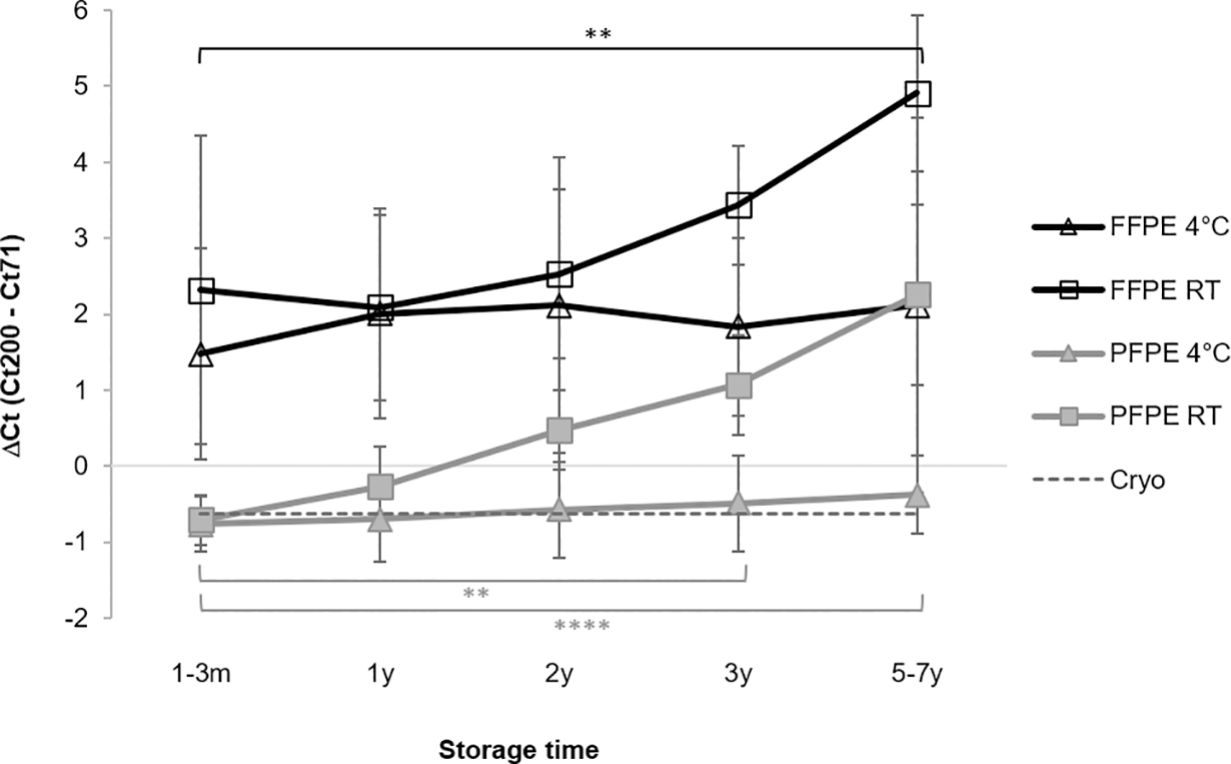
Improved RT-qPCR-performance of RNA from human tissue blocks stored at 4°C. RT-qPCR-performance of RNA from human FFPE and PFPE tissues stored prior to RNA extraction at 4°C or at room temperature (RT) for up to 7 years. Mean delta Ct values with standard deviation of two assays with different amplicon lengths of human GAPDH gene (Ct 200 bp–Ct 71 bp) from up to 8 cases are shown. Statistically significant differences (P < 0.05) between the first (1-3m) and following timepoints of RT storage are shown. No significant differences were found in case of storage at 4°C.

This storage effect was further investigated by calculating mean delta Ct values for a short (71 bp) and a longer fragment (200 bp) from all the 8 cases stored for up to seven years (Fig 5). Overall, delta Ct values were higher for FFPE compared to PFPE, indicating decreased RT-PCR efficiency for longer fragments. In both FFPE and PFPE tissues delta Ct increased over storage time in case of PET blocks stored at room temperature but remained constant in case of blocks stored at 4°C (Fig 5).

**Fig 6:**
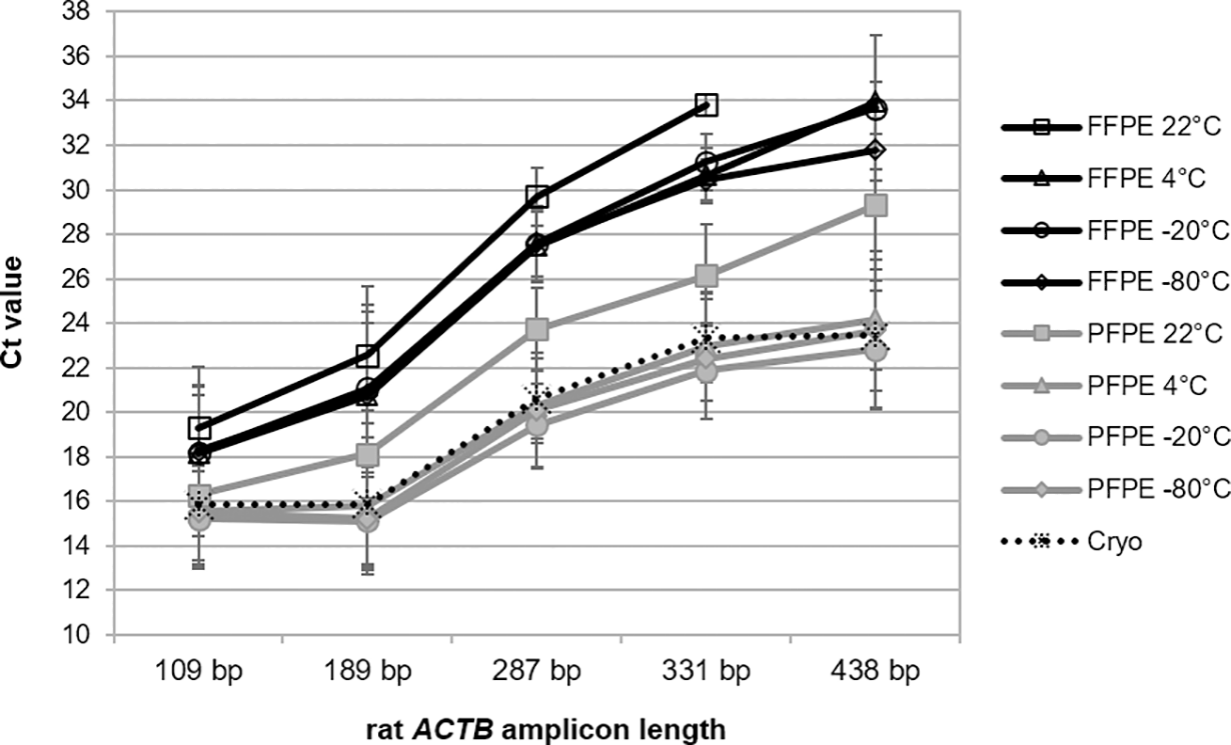
Improved RT-qPCR-performance of RNA from rat tissue blocks stored at 4°C or frozen for 9 years. PFPE and FFPE tissues were stored 108 months (m) at different temperatures (22°C, 4°C, -20°C, and -80°C). Reverse transcription and amplification in five different rat *ACTB* SYBR-Green RT-qPCR assays ranging from 109 to 438 base pairs (bp). Mean Ct values with standard deviation from triplicate extractions from each of five different tissues (liver, kidney, spleen, lung and intestine) are shown for each assay. RNA from cryo preserved tissue was used as a reference, shown as dashed line.

In the animal model RNA isolated from fresh, untrimmed PET blocks stored for nine years were investigated in a series of SYBR-Green assays for amplification of *ACTB* fragments with increasing amplicon lengths ranging from 109 to 438 bp (Fig 6). As a reference Ct values were generated with RNA from cryo-preserved tissues. In concordance to the results achieved with human tissues, a clear storage temperature effect became visible for RNA isolated from both FFPE and PFPE tissues. RNA from PET blocks stored at lower temperature, ranging from 4 to -80°C, demonstrated superior qPCR efficiency compared to the corresponding tissues stored at 22°C. Increase in Ct values for tissues stored at 22°C compared to tissues stored at lower temperature were between 1.5 to 4 Ct in case of FFPE and between 0.5 to 6 in case of PFPE tissues depending on the amplicon length. However, overall RT-qPCR efficiency from PFPE tissues stored at 22°C was still higher (i.e., lower delta Ct) compared to RNA from FFPE tissues stored frozen or at 4°C. Moreover Ct values from PFPE tissues stored at lower temperatures were similar to the Ct values from corresponding cryo-preserved tissues. In contrast, differences in Ct values between PFPE tissues stored at 22°C and cryo-preserved tissues increased with amplicon lengths from 0.5 Ct for the smallest (109 bp) to up to 6 Ct for the largest (438 bp) fragment. Differences between Ct values from FFPE tissues and cryo-preserved tissues were in the range of 2–3 for the smallest and up to 10 Ct for the largest fragment.

### Storage of PET blocks at lower temperatures prevents from DNA degradation

**Fig 7:**
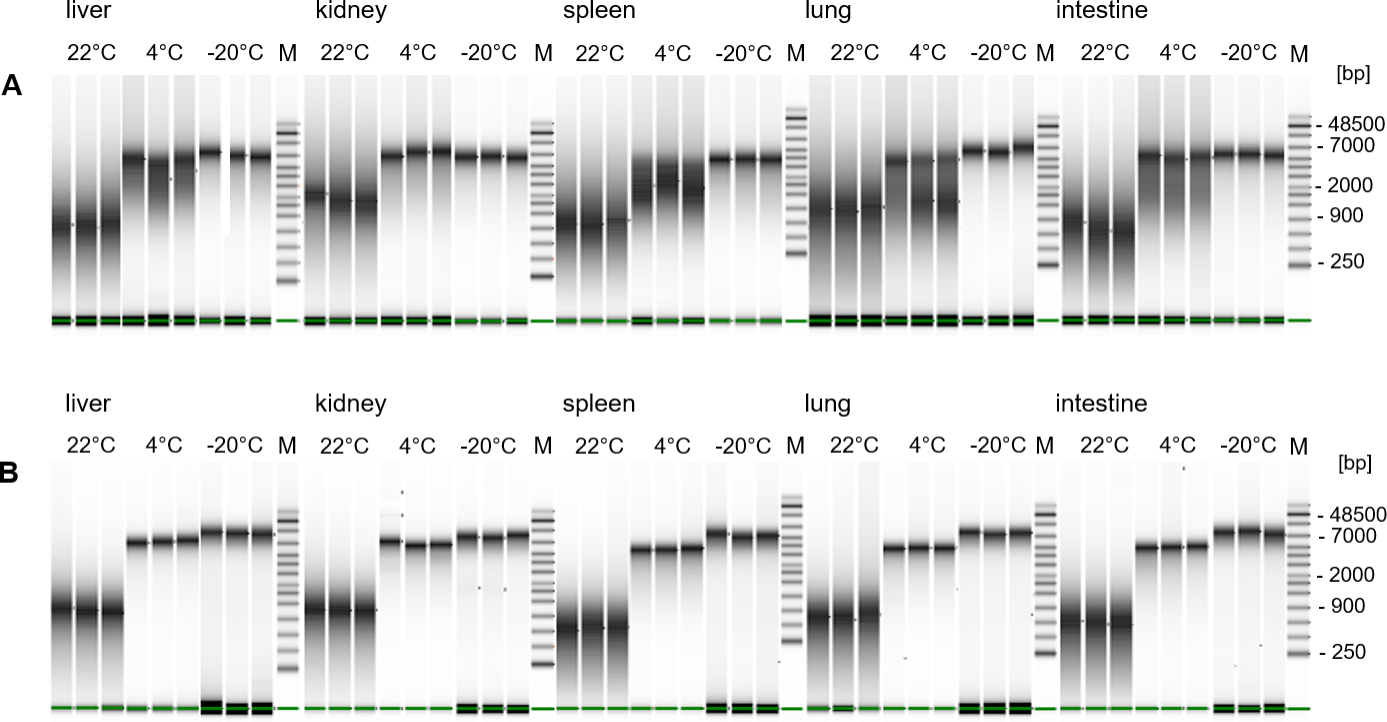
Storage of PET blocks for 9 years at lower temperatures prevents from DNA degradation. Analysis of DNA integrity from FFPE (A) and PFPE (B) tissues of rat liver, kidney, spleen, lung and intestine on Agilent 4200 TapeStation system with genomic DNA Analysis ScreenTape assay. PET blocks were stored prior to DNA extraction for 108 months at 22°C, 4°C, and -20°C. DNA was extracted from 3x 10μm sections in triplicates.

Integrity of DNA isolated from rat FFPE and PFPE tissues stored at different temperatures for 9 years was assessed on a TapeStation (Fig 7). Differences in DNA integrity depending on storage temperature were found for both FFPE and PFPE tissues. Genomic DNA extracted from PET blocks stored for 9 years at 22°C was highly degraded, appearing as a smear in the range of 0.3 to 1.5 kb (Fig 7 and Table 1), with no high-molecular weight band visible across all analyzed tissues. PET block storage at 4°C resulted in improved DNA quality, less fragmentation and in most cases distinct bands of 4.4 to 5 kb became visible. Highest integrity with least fragmentation was received from PET blocks stored frozen at -20°C with a distinct DNA band of around 5 to 7 kb. DNA isolated from FFPE tissues stored at 4°C showed more DNA fragmentation than PFPE tissues.

**Table 1:** Average DNA molecule size from rat PET blocks stored at different temperatures for 9 years.

	FFPE			PFPE		
	Size [bp]	From [bp]	To [bp]	Size [bp]	From [bp]	To [bp]
**22°C**	1106	1037	1342	695	340	1512
**4°C**	4417	2615	14232	4909	2323	11630
**-20°C**	5075	2373	16353	7701	2787	23896

Main peak and range of DNA sizes in base pairs (bp). Main peak and range of DNA sizes in base pairs (bp) calculated with Agilent 4200 TapeStation system using the genomic DNA Analysis ScreenTape assay. Average values are shown for three replicates from five different tissue types each.

### Improved qPCR-performance of DNA from PET blocks stored at lower temperatures

**Fig 8:**
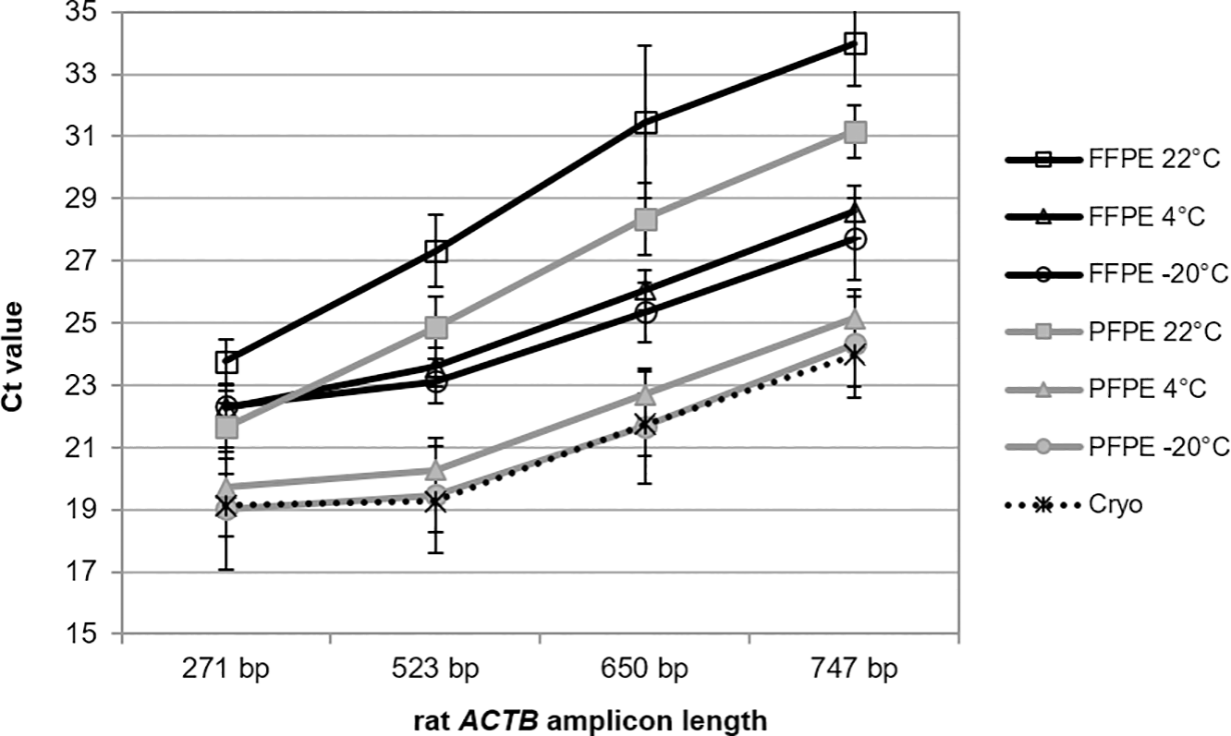
Improved qPCR-performance of DNA from PET blocks stored for 9 years at lower temperatures. FFPE and PFPE animal tissues were stored prior to DNA extraction for 108 months at different temperatures (22°C, 4°C, and -20°C). qPCR was performed with four different SYBR-Green qPCR assays of the rat *ACTB* gene with amplicon length of 271, 523, 650 and 747 base pairs (bp). Triplicate extractions from each PET block were amplified. DNA from cryo-preserved rat tissue was used as reference, shown as dashed line. Mean Ct values with standard deviation from triplicate extractions from each of five different tissues (liver, kidney, spleen, lung and intestine) are shown for each assay.

qPCR-performance of DNA isolated from rat PET blocks stored for nine years at 22, 4 or -20°C was tested using amplicons of the *ACTB* gene with different lengths of 271, 523, 650 and 747 bp (Fig 8). A storage temperature effect was observed for both FFPE and PFPE tissues. Similar to RT-qPCR with human and animal tissues overall amplification efficiency was higher in case of DNA from PET blocks stored at lower temperature (4 or -20°C) compared to storage at 22°C. DNA isolated from PFPE tissues stored at -20°C demonstrated identical and from blocks stored at 4°C only marginally inferior qPCR-performance compared to DNA isolated from the cyro-references. Due to the negative impact of chemical modification by formaldehyde consistent higher Ct values were reached in case of DNA from FFPE tissues compared to PFPE tissues. The difference between corresponding FFPE and PFPE tissues stored at 4 or -20°C was about 4 Ct, and for tissues stored at 22°C about 2–3 Ct. These differences remained constant independent from amplicon length.

## Discussion

Many studies were published on RNA expression analyses from human archived FFPE tissues [32–35] and on the impact of RNA degradation or low quality RNA from FFPE tissue on gene expression profiling [36,37]. Furthermore time-dependent degradation of RNA in PET blocks was documented before [38] and few studies investigated the effect of storage conditions on RNA integrity. Von Ahlfen et al. [3], using a rat tissue animal model, found increased RNA degradation in FFPE tissues stored for 12 months at room temperature versus storage at 4°C but did not investigate the effect on downstream applications such as RT-qPCR. Kokkat et al. [39] did not find significant differences for RNA integrity between macromolecules processed from FFPE tissues stored for up to 12 years compared to blocks less than one year old. However, in their study RNA quality from FFPE clinical tissues was extremely low throughout all time points investigated, starting with RQI (RNA quality indicator) numbers of 1.9–2.6 at the beginning of the study. It seems unlikely that within such low integrity level, degradation kinetics can be monitored by capillary electrophoresis. In addition in this study no downstream performance data were presented.

It is well known that preanalytical handling steps such as cold ischemia time (time between resection and fixation), fixative compositions and volume, fixation time, tissue size, transport conditions, and processing can influence bioanalyte quality and the outcome of analytical results [40–43]. Within the EU FP7 project SPIDIA evidence-based guidelines were developed for standardization and improvement of pre-analytical procedures for in vitro diagnostics. These guidelines were the basis for technical specifications (TS) recently published by the European Committee for Standardization (Comité Européen de Normalisation, CEN) with recommendations for handling, documentation and processing of FFPE tissue specimens during the preanalytical phase intended for RNA, protein and DNA analysis [44–46]. The study presented here, was started within SPIDIA and special emphasis was laid on preanalytical handling steps as meanwhile outlined in the respective CEN/TS. This included minimizing cold ischemia times, use of NBF with controlled pH and with a ratio of fixative to tissue volume of at least 10:1, thorough grossing, strictly controlled fixation time and use of controlled processors with regular replacement of all reagents. In case of fixation with PAXgene Tissue recommendations of the manufacturer for fixation, stabilization and processing was followed. In order to avoid cross contamination of PAXgene Tissue fixed samples with formaldehyde, a washing step of formalin-fixed samples in 70% ethanol was performed prior further processing and embedding of PAXgene and NBF treated tissues. This preanalytical handling assured the highest possible RNA quality from both FFPE and PFPE tissues at the beginning of the storage study with RIN values in the range of 2–7 in case of human and 4–9 in case of rat tissues. The high RNA quality at the beginning of the study allowed us to determine the dynamic effect of PET storage temperature on RNA integrity and performance in RT-qPCR over a storage time of up to nine years. The advantage of the animal model was that workflow parameters which are difficult or impossible to control in clinical routine such as warm and cold ischemia could be narrowed down. This resulted in even higher RNA integrity compared to the human tissues. In addition the animal model allowed grossing of as many corresponding tissues per tissue type as needed to extend the storage conditions to frozen temperatures.

Just recently a study was published which investigated the effect of seven years storage at room temperature on RNA and microRNA stability in PFPE human tissues [47]. Sanchez et al. found a median reduction in RIN values from 3.6 to 2.0 and increased Ct value of 2.5 for a GAPDH fragment, which is in line with our findings. However they did not include paired samples of Formalin and cryo-preserved tissues and did not investigate the effect of storage temperature in their study. Actually, to our knowledge we are the first to systematically investigate in a long-term storage study the impact of different fixatives and storage conditions on nucleic acid integrity and usability in archived human and animal PET blocks.

In case of human samples different fixation times were applied to simulate a realistic scenario of the routine workflows of pathology labs. No adverse effect of over- or under-fixation was observed by RIN analysis. In contrast using the fragment length qRT-PCR assay an impact of fixation time was observed for FFPE samples in a previous study [7]. Therefore, for PCR analysis of storage effects only samples fixed for 24 hours (except for case 6 with 3 hours fixation time) samples were analyzed. However, both scenarios, human and animal model, led to similar results. In all PET blocks investigated (FFPE and PFPE, rat and human tissue) RNA and DNA degraded over time depending on the storage temperature, with similar degradation kinetic. In the case of RNA when stored at room temperature degradation started immediately and RNA integrity numbers (RIN) reached its lowest level after 6–12 months. Further degradation below RIN 2–3 could not be detected. Storage at 4°C slowed down but did not prevent degradation, lowest average RIN values of around 3 were reached after 24 to 36 months. Freezing and storage at -20°C prevented RNA in PETs from degradation. Storage at even lower temperature of -80°C did not seem to gain any additional value with regard to nucleic acid integrity or usability in downstream applications but led to cracks within the paraffin blocks which impeded sectioning and might even hinder further morphological analyses.

Chemical modification during formaldehyde fixation causes nucleic acid degradation [5]. However, little is known about mechanism and kinetic of RNA degradation in PET. At least in case of FFPE tissue, enzymatic degradation seems to be unlikely since fixation with formalin irreversibly inactivates RNases. This is also supported by the observation that DNA degrades in PET blocks according to storage temperature. DNase is unlikely to survive processing with multiple alcohol incubation steps and therefore cannot be involved anymore in degradation. Instead chemical degradation by oxidation and hydrolysis, triggered by residual water trapped in the tissue due to incomplete processing, might be involved. Von Ahlfen et al. [3] speculated that embedding conditions like incubation time and temperature in hot paraffin influence integrity. Evers et al. [41] demonstrated that the paraffin embedding step contributes to RNA damage and thus recommended to minimize time and temperature that tissues spend in warm hydrocarbon solvents during processing. Chung et al. [42] suggested that complete removal of water during processing and paraffin embedding is required and Xie et al. [48] reported that insufficient replacement of endogenous water due to shortened processing time correlates with protein degradation and reduced immunoreactivity during storage. However, as shown in this study, even under optimal processing conditions degradation of biomolecules over time cannot be prevented.

In the archives of pathology departments and biobanks FFPE blocks from daily routine are stored at room temperature in large quantities. Morphological details can be analyzed from FFPE blocks, which were stored over decades. Also molecular analyses are possible to some degree, but analytes when burdened with chemical modifications through formaldehyde fixation, may lead to DNA sequence alterations, and wrong quantification [6,49]. Due to crosslinking and chemical modification of RNA from FFPE tissue the reverse transcription step in RT-qPCR is impeded relative to amplicon lengths indicated by increasing Ct values compared to a cryo-preserved reference [15]. With this regard the advantage of using chemically unmodified nucleic acids in PCR became also visible in this study. PCR performance was improved by magnitudes with nucleic acids from PFPE versus FFPE tissues.

While storage of PETs at 4°C did not prevent nucleic acids from degradation, in both, human and rat, tissues, RT-qPCR and qPCR efficiency was remarkably unaffected from storage time. In case of RNA and DNA from PFPE tissues stored at 4°C for up to nine years, performance was close or even equivalent to nucleic acids from cryo-preserved controls independent from amplicon lengths. In case of RNA and DNA from FFPE tissues storage at lower temperatures could not prevent decreased PCR efficiency. This became evident from overall higher Ct values, which correspond to lower numbers of target gene fragments accessible by PCR, compared to PFPE tissues and cryo-references. However, this adverse effect did not increase over storage time. In RT-qPCR the differences in Ct values, i.e. the number of target gene fragments, between a long and a short amplicon remained more or less constant over time with RNA from PET blocks stored at 4°C. In contrast this delta Ct value increased with storage time in case of RNA from FFPE and PFPE tissues stored at room temperature (Fig 5).

These observations also confirm that quality assessment of RNA from FFPE tissue by electrophoresis or spectrophotometry and deduced quality scores i.e. RIN values are of limited use as a predictive value for RT-qPCR efficiency, as published earlier [7,15].

## Conclusions

Developments in personalized medicine may require re-analysis of tissue that has been obtained from a patient several months to years ago using recent molecular techniques. As demonstrated here storage at room temperatures in routine archives leads to a loss of nucleic acid quality from PET blocks. The observed constant deterioration of quality might lead to PET blocks unusable for research applications or diagnostic retesting at some point. One possibility to decelerate this process is storage at lower temperatures. Further improvement is possible by the use of a formaldehyde free fixative such as PAXgene Tissue, or ideally a combination of both. For cost reasons use of formaldehyde alternatives and cooled storage of all tissue samples accumulating in a routine pathology laboratory might not be realistic. Nevertheless, for prospective studies whenever maintaining high quality molecular information is needed, use of formaldehyde free fixatives and storing PET blocks at 4°C or freezing should be considered. Ongoing studies have to show the impact of long term storage at different temperatures on next generation sequencing quality parameters.

## Supporting information

S1 Table Human clinical cases overview (m = malignant, nm = non-malignant). (XLSX)

S2 Table Correlation between increase in Ct value and decrease in target gene fragments accessible for PCR, assuming optimal PCR efficiency. (XLSX)

S3 Table RIN and Ct value from human and rat tissue samples. (XLSX)
